# Modeling the potential of *w*Au-*Wolbachia* strain invasion in mosquitoes to control *Aedes*-borne arboviral infections

**DOI:** 10.1038/s41598-020-73819-1

**Published:** 2020-10-08

**Authors:** Samson T. Ogunlade, Adeshina I. Adekunle, Michael T. Meehan, Diana P. Rojas, Emma S. McBryde

**Affiliations:** 1 Australian Institute of Tropical Health and Medicine, James Cook University, Townsville, QLD Australia; 2 College of Medicine and Dentistry, James Cook University, Townsville, QLD Australia; 3 College of Public Health, Medical and Veterinary Sciences, Division of Tropical Health and Medicine, James Cook University, Townsville, QLD Australia

**Keywords:** Applied mathematics, Viral infection

## Abstract

Arboviral infections such as dengue, Zika and chikungunya are fast spreading diseases that pose significant health problems globally. In order to control these infections, an intracellular bacterium called *Wolbachia* has been introduced into wild-type mosquito populations in the hopes of replacing the vector transmitting agent, *Aedes aegypti* with one that is incapable of transmission. In this study, we developed a *Wolbachia* transmission model for the novel *w*Au strain which possesses several favourable traits (e.g., enhanced viral blockage and maintenance at higher temperature) but not cyctoplasmic incompatibility (CI)—when a *Wolbachia*-infected male mosquito mates with an uninfected female mosquito, producing no viable offspring. This model describes the competitive dynamics between *w*Au-*Wolbachia*-infected and uninfected mosquitoes and the role of imperfect maternal transmission. By analysing the system via computing the basic reproduction number(s) and stability properties, the potential of the *w*Au strain as a viable strategy to control arboviral infections is established. The results of this work show that enhanced maintenance of *Wolbachia* infection at higher temperatures can overcome the lack of CI induction to support *w*Au-*Wolbachia* infected mosquito invasion. This study will support future arboviral control programs, that rely on the introduction of new *Wolbachia* variants.

## Introduction

Arthropod-borne viruses, or arboviruses, are viruses that are transmitted via blood feeding arthropods^1^. Arboviral infections such as dengue, Zika and chikungunya are fast spreading diseases that pose significant health problems globally^2–5^. These viral infections, in particular dengue, are transmitted mainly by* Aedes aegypti* and sometimes by *Aedes albopictus* (Asian Tiger) female mosquitoes when taking a blood meal from the host^6,7^. Approximately 390 million dengue infections are estimated to occur worldwide annually, putting 40% of the total human population at risk^8^. Dengue infection is the most geographically wide-spread of the arboviral infections^3,8^. It has different severity levels which are classified according to disease progression from dengue without warning signs to dengue with warning signs and then severe dengue^9^. Clinical manifestation includes sudden high-grade fever, headache, nausea, arthralgia, eye pain, muscle ache and rash in some cases^10^. Presently, there is no specific universal treatment for dengue infections: the vaccine envelopment targets young populations; the efficacy of the only vaccine licensed depends on prior immunity to at least one serotype of dengue; and it provides heterogeneous protection against the different serotypes^11,12^.

Other arboviral infections such as Zika, chikungunya and yellow fever are also of global health concern^13^. These arboviral infections have occurred simultaneously with dengue^13,14^. Some of these infections share many similar clinical manifestations with dengue infection and also allow arboviral coinfection such as dengue and chikungunya^15^, chikungunya and Zika^16^ and yellow fever and chikungunya^17^. Although, there are no specific treatments for Zika and chikungunya viral infections, these infections can be managed by supportive treatment of symptomatic individuals and adequate rest. This treatment includes fluid intake and administering drugs such as acetaminophen to suppress pain and fever^18,19^. However the prevention strategy for yellow fever infection is available i.e. vaccination^20,21^.

To control these infections, an intracellular bacterium called *Wolbachia* can be used to suppress transmission in arthropods such as mosquitoes and flies^22–25^. *Wolbachia* infection inhibits arboviral transmission in mosquitoes via four mechanisms: immune priming—preactivation of the mosquito immune system; induction of the phenoloxidase cascade—triggers immune response to viruses; competition of intracellular resources—inducing authophagy; and induction of microRNA-dependent immune pathways—essential for gene regulation and stability, immune defense, ageing and organ differentiation^26^. This endosymbiotic bacterium which exists naturally in more than 50% of all insect species can be found within the cytoplasm of the cells of their hosts^25,27,28^. Whilst *Wolbachia* is not naturally present in *Aedes aegypti*, it can be introduced via stable transinfections using microinjections^29,30^.

The *Wolbachia*-based control strategy is carried out by infecting mosquitoes with a strain of *Wolbachia* and then releasing them into wild mosquito populations in the hopes of replacing the vector transmitting agent *Aedes aegypti* with one that is incapable of transmission^29–31^. Infecting an *Aedes* mosquito with *Wolbachia* can change some of the *Aedes* characteristic features. In practice, *Wolbachia* can reduce the life-span of mosquitoes by half producing a deleterous fitness effect^32^. Another feature is cytoplasmic incompatibility (CI)^22,33–35^ which occurs when a *Wolbachia* infected male mates with an incompatible female mosquito (usually *Wolbachia* uninfected) producing no offspring^36^. Other features of *Wolbachia* which serve as liabilities in mosquitoes include: imperfect maternal transmission (IMT)^30,37^ and loss of *Wolbachia* infection (LWI). LWI impedes the establishment of *Wolbachia*-infected mosquitoes and is a result of mosquito vulnerability to high temperature^38,39^.

However, a novel strain of *Wolbachia*: *w*Au, has shown to produce high viral blockage whilst maintaining *Wolbachia* infection in *Aedes* mosquitoes at higher temperature^23^. Moreover, *w*Au allows superinfection to occur when *w*Au and other strains of *Wolbachia* co-exist in the vector host^23^. Despite these favourable features, *w*Au does not induce CI^23^. Although CI absence does not establish *Wolbachia* infected mosquitoes, the effect could be outweighed by LWI and IMT^37^.

The difference in the common *Wolbachia* strain features are described in Table 1 below.

**Table 1:** Characteristics of different *Wolbachia* strains in *Aedes* mosquitoes: as defined in^22^, the percentages (%) of the effects of these features are: High$\longrightarrow$ above 90, Medium$\longrightarrow$ 20 to 90, Low$\longrightarrow$ less than 20 and None$\longrightarrow$ 0, (features not detected).

Features	wAu	wMel	wMelPop	wAlbA	wAlbB
Viral blockage	High^23^	Medium^40,41^	High^29,41–43^	Medium^23^	High^44^
Maternal transmission	High^23^	High^30^	High^32,45^	High^23^	High^46^
Loss of *Wolbachia* infection at higher hemperature	Low^23^	High^23^	High^23^	Medium^23^	Medium^23^
Fitness cost	Medium^23^	Medium^23^	High^47,48^	High^23^	Medium^22^
Cytoplasmic incompatibility	None^23^	High^30^	High^32,45^	High^23^	High^46^

In general, the introduction of mathematical models to understand infection dynamics of diseases has long been helpful in the area of disease control^49^. A number mathematical models of *Wolbachia* dynamics in a mosquito population have been formulated^37,50–58^. Some of these models introduced *Wolbachia* strain(s) into a mosquito population and classified them into age-sturctured *Wolbachia*-infected and -uninfected mosquito compartments^37,53,54,57^. Ndii et al.^53^, formulated a mathematical model for the *Wolbachia* interaction between the immature stages (aquatic stage), adult male and female mosquito populations to investigate the persistence of mosquitoes infected with *Wolbachia* when competing with the uninfected ones. They derived the steady state solutions and showed that parameters such as maternal transmission, reproductive, death and maturation rates drive the persistence of the *Wolbachia*-infected mosquito population. A similar model developed by Xue et al. considered the *Wolbachia*-induced fitness change and the CI effect^57^. They showed that if the basic reproduction number ($R_0$) of the *Wolbachia*-infected mosquitoes is less than one, an endemic *Wolbachia* infection can still occur via backward bifurcation if a sufficient number of the mosquitoes are introduced into the population. A mathematical model of *Wolbachia* to control dengue fever transmission^52^ was developed by Hughes et al. The model showed that the use of *Wolbachia* has high potential to control dengue where the $R_0$ due to *Wolbachia*-infected *Aedes* mosquitoes is not too large in endemic areas. Another study of a *Wolbachia* invasive model incorporated IMT and LWI and showed that CI does not guarantee the establishment of *Wolbachia*-infected mosquitoes as the disadvantages derived from IMT and LWI in the production of *Wolbachia*-infected mosquitoes could outweigh CI^37^.

Additionally, a study conducted by O’Reilly et al combining multiple modeling methods, was used to estimate the burden of dengue and map its distribution across Indonesia^59^. They predicted that there was a reduction in dengue transmission after a nationwide release of *w*Mel-*Wolbachia*-infected mosquitoes. In addition, they predicted about 86% of the estimated 7.8 million annual cases of symptomatic dengue in Indonesia could be averted following a complete nationwide rollout of *Wolbachia*-infected mosquitoes. Recently, a modeling study presented a dengue transmission model in the presence of female wild-type and *w*MelPop *Wolbachia*-infected *Aedes aegypti* mosquitoes. They concluded that although the *w*MelPop strain reduces the lifespan of infected mosquitoes, which could be challenging to achieve replacement of wild-type mosquitoes, its optimal release ensured the replacement of wild-type mosquitoes and also reduced dengue burden in the human population^51^. A mosquito-*Wolbachia* model was developed by Xue et al, to compare the potential effectiveness of two *Wolbachia* strains (*w*Mel and *w*AlbB) to control arboviral spread^60^. They observed that each of the two different strains of *Wolbachia* can effectively decrease the rate of arboviral transmission.

Here, we develop a general *Wolbachia* model capable of faithfully replicating all of the strain features described in Table 1. The general transmission model is an extention of the *Wolbachia* transmission model introduced in Adekunle et al.^37^, which described the competitive dynamics between (*w*Mel-like) *Wolbachia*-infected and uninfected mosquitoes. Despite the non-induction of CI in *w*Au-*Wolbachia*-infected mosquitoes, *w*Au infection is retained and able to block viral transmission efficiently compared to other strains even at high temperature. Therefore, we incorporated this feature to determine if the advantages (*Wolbachia* retainment) of the *w*Au strain outweigh the ineffectiveness of CI. This feature has not been considered in previous models. Furthermore, we incorporate imperfect maternal transmission into the model. By analysing the system via computing the basic reproduction number(s) and investigating the stability properties of the equilibrium points, the potential of the *w*Au strain as a viable strategy to control *Aedes*-borne infections can be established. The aim of this modeling approach is to support future *Aedes*-borne viral control programs, particularly with the introduction of new *Wolbachia* variants.

## Methods

### Model formation

Here, we investigate a modified *Wolbachia* transmission model studied in Adekunle et al.^37^, focusing on a novel *Wolbachia* strain, *wAu*, which has high retainment, high viral blockage and does not induce CI. The mosquito population is subdivided into two groups: the uninfected mosquitoes $(.)_u$ and the *Wolbachia* infected mosquitoes $(.)_w$. The term (.) can be aquatic/immature (eggs, larvae and pupae) *A*, male *M* or female *F* mosquitoes. In addition, we denote the aquatic/immature stages, mature male and mature female uninfected mosquitoes as $A_u$, $M_u$, $F_u$, and *Wolbachia*-infected mosquitoes as $A_{w}$, $M_{w}$, $F_{w}$ respectively. As in Adekunle et al.^37^ the model also incorporates the IMT of *w*Au-*Wolbachia*.

There are four possible mosquitoes’ mating pairs: $F_uM_u$, $F_uM_w$, $F_wM_u$ and $F_wM_w$. As *Wolbachia* infection is maternally transmitted, $F_uM_u$ and $F_uM_w$ will produce uninfected offspring while $F_wM_u$ and $F_wM_w$ will typically produce infected offspring. However if there is imperfect maternal transmission, the two latter strategies could produce some proportions of uninfected offspring^23^.

**Figure 1:**
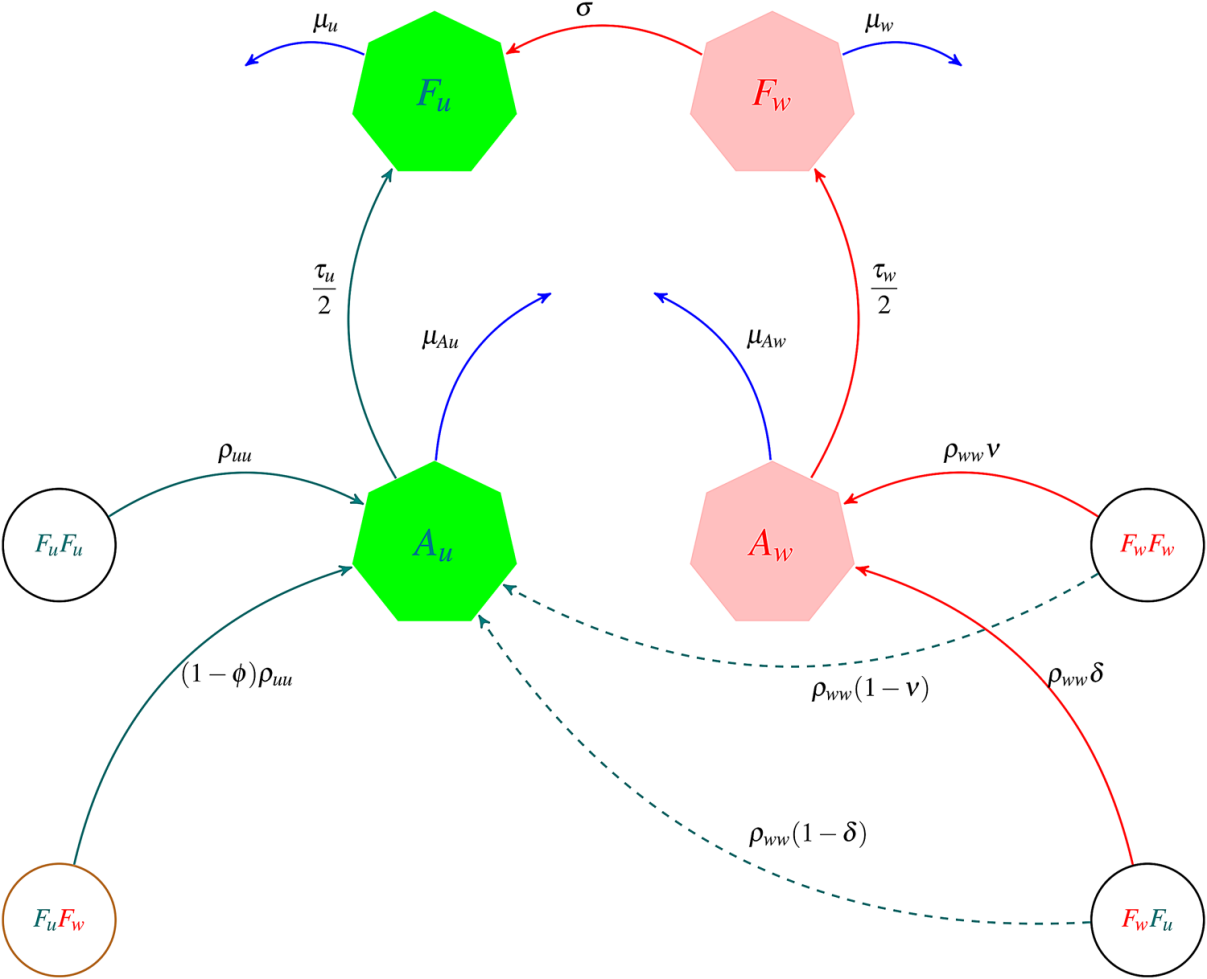
General model showing the *Wolbachia* infection dynamics in mosquitoes as *M* has been set equal to *F*. The green and pink compartmental polygons represent wild-type and *Wolbachia*-infected mosquitoes respectively. $$A_{u}$$ and $$F_{u}$$ represent the aquatic (eggs, larvae and pupae) and adult female mosquitoes for the uninfected mosquito population respectively while $$A_{w}$$ and $$F_{w}$$ represent their *Wolbachia* infected counterparts. The teal and red arrows illustrate the population progression of uninfected and *Wolbachia*-infected mosquitoes respectively. The four circles (three black and one brown) represent the mosquito mating strategies. The effect of cytoplasmic incompatibility ($$\phi $$), i.e. for *w*Au and *w*Mel strains, $$\phi =0$$ and $$\phi =1$$ respectively, is illustrated by the brown-circled $$F_{u}$$$$F_{w}$$. The dashed lines represent the proportion of uninfected offspring caused by imperfect maternal transmission (IMT). The blue lines depict mosquito mortality. If there is loss of *Wolbachia* infection (LWI), $$\sigma > 0$$. But if there is no LWI as in *w*Au-*Wolbachia* strain, then $$\sigma =0$$.

To mathematically write the system of differential equations governing the *Wolbachia* transmission dynamics, we express the feasible mating strategies of uninfected and *Wolbachia* infected mosquito populations together with their per capita egg laying rates as Eqs. (1)–(6):

1 $$\begin{aligned} \frac{dA_{u}}{dt}= & {} \left[ \frac{\rho _{uu} (F_u M_u + (1-\phi ) F_u M_{w}) + \rho _{ww}( (1-\delta ) F_{w}M_u + (1-\nu ) F_{w}M_{w})}{M}\right] \left( 1-\frac{A}{K} \right) \nonumber \\&\qquad -(\tau _{u} +\mu _{Au}) A_u, \end{aligned}$$

2 $$\begin{aligned} \frac{dF_{u}}{dt}= & {} (1-\psi )\tau _{u}A_u +\sigma F_w -\mu _u F_u, \end{aligned}$$

3 $$\begin{aligned} \frac{dM_{u}}{dt}= & {} \psi \tau _{u}A_u +\sigma M_w -\mu _u M_u \end{aligned}$$

4 $$\begin{aligned} \frac{dA_{w}}{dt}= & {} \left[ \frac{\rho _{ww}(\nu F_{w}M_{w} + \delta F_{w}M_u)}{M}\right] \left( 1-\frac{A}{K} \right) - (\tau _{w} +\mu _{Aw}) A_{w}, \end{aligned}$$

5 $$\begin{aligned} \frac{dF_{w}}{dt}= & {} (1-\psi )\tau _{w}A_{w} -\sigma F_w- \mu _{w} F_{w}, \end{aligned}$$

6 $$\begin{aligned} \frac{dM_{w}}{dt}= & {} \psi \tau _{w}A_{w} -\sigma M_w - \mu _{w}M_{w}, \end{aligned}$$

where $$F=F_u +F_{w}$$, $$M=M_u +M_{w}$$, $$A=A_u +A_{w}$$.

Here, $\phi$ represents the CI effect which can be either 0 if there is no CI, or 1 if CI is present. $\sigma$ is the effect of LWI, such that it can either be 0, if there is no *Wolbachia* loss or greater than zero otherwise. In Adekunle et al.^37^ where CI is assumed and LWI is considered, these quantities are set to $\phi =1$ and $\sigma \ge 0$. In our modified model, considering different strains with the exception of *w*Au strain, $\phi =1$ and $\sigma$ could vary from values greater than zero onwards. However, for the *w*Au-*Wolbachia* strain, CI is ineffective and high retainment of *w*Au-*Wolbachia* infection even at high temperatures^23^ is established, therefore we set $\phi =0$ and $\sigma =0$. Our model also incorporates imperfect maternal transmission generating a proportion of infected and uninfected offspring from mating of both $F_w M_{u}$ and $F_w M_{w}$ mosquitoes. To simplify the system, we assume that $M=F$ in accordance with the observed ratio of male to female mosquitoes of 1.02:1^62^. That is, we set $\psi = 1/2$ (Fig. 1). By this, it follows that the system of ordinary differential equations (ODEs) in Eqs. (1)–(6) can be reduced to (7)–(10) which is the governing *Wolbachia* infection dynamics.

To mathematically express the above schematics, we have that, the feasible mating strategies of uninfected and *Wolbachia* infected mosquito populations together with their per capita egg laying rates are given by the following differential system:

7 $$\begin{aligned} \frac{dA_{u}}{dt}= & {} \left[ \frac{\rho _{uu} (F_u^2 + (1-\phi )F_u F_{w}) + \rho _{ww}((1-\nu ) F_{w}^2 +(1-\delta )F_w F_{u})}{F}\right] \left( 1-\frac{A}{K} \right) -(\tau _{u} +\mu _{Au}) A_u, \end{aligned}$$

8 $$\begin{aligned} \frac{dF_{u}}{dt}= & {} \frac{\tau _{u}}{2}A_u +\sigma F_w-\mu _u F_u, \end{aligned}$$

9 $$\begin{aligned} \frac{dA_{w}}{dt}= & {} \left[ \frac{\rho _{ww} (\nu F_{w}^2 +\delta F_w F_{u})}{F}\right] \left( 1-\frac{A}{K} \right) - (\tau _{w} +\mu _{Aw}) A_{w}, \end{aligned}$$

10 $$\begin{aligned} \frac{dF_{w}}{dt}= & {} \frac{\tau _{w}}{2}A_{w} - \sigma F_w- \mu _{w} F_{w}, \end{aligned}$$

where $F=F_u +F_{w}$ and $A=A_u +A_{w}$. Before proceeding, we rescale each of our state variables according to the maximum total population size, which by Adekunle et al., 2019^37^ is set by

$$\begin{aligned} A_u(t) +F_u(t)+A_{w}(t) +F_{w}(t)\le & {} K + \dfrac{\tau _u K}{2\mu _u} + \dfrac{\sigma \tau _w K}{2\mu _u(\mu _w +\sigma )} + \dfrac{\tau _w K}{2(\mu _w +\sigma )}\\\le & {} K\left( 1+\dfrac{1}{2}\left( \dfrac{\tau _u}{\mu _u} + \dfrac{\tau _w}{(\mu _w +\sigma )}\left( 1+\dfrac{\sigma }{\mu _u}\right) \right) \right) \\\le & {} \alpha K \end{aligned}$$

where $$\alpha = 1+\frac{1}{2}\left( \frac{\tau _u}{\mu _u} + \frac{\tau _w}{(\mu _w +\sigma )}\left( 1+\frac{\sigma }{\mu _u}\right) \right) $$.

The closed set

$$\Omega = \left\{ (A_u, F_u, A_w, F_w) \in \mathbb {R}_+^4 \, | \, A_u + F_u + A_w + F_w \le \alpha K \right\} $$

which is a feasible region for the above system dynamics is positively invariant^37^.

Hence, we let $\bar{A}_u = \frac{A_u}{\alpha K}$, $\bar{A}_w = \frac{A_w}{\alpha K}$, $\bar{F}_u = \frac{F_u}{\alpha K}$, $\bar{F}_w = \frac{F_w}{\alpha K}$, $\bar{A}=\bar{A}_u +\bar{A}_{w}$ and $\bar{F}=\bar{F}_u +\bar{F}_{w}$. Also, letting $\nu =1$, we assume a perfect maternal transmission for the reproduction outcome of $\bar{F}_w \bar{M}_{w}$ mating. Therefore, the general *Wolbachia* model in terms of population proportions is given by Eqs. (12)–(14). Hereafter it is clear that we refer to the scaled values of each state variable and as such drop the overbar from our notation. The scaled model below now evolves in the feasible region $\bar{\Omega }$, where $\bar{\Omega } = \left\{ (\bar{A}_u, \bar{F}_u, \bar{A}_w, \bar{F}_w) \in \mathbb {R}_+^4 \, | \, \bar{A}_u + \bar{F}_u + \bar{A}_w + \bar{F}_w \le 1 \right\}$.

11 $$\begin{aligned} \frac{d\bar{A}_u}{dt}= & {} \left[ \frac{\rho _{uu} (\bar{F}_u^2 + (1-\phi )\bar{F}_u \bar{F}_w) + \rho _{ww}(1-\delta )\bar{F}_w \bar{F}_u}{\bar{F}}\right] \left( 1-\alpha \bar{A} \right) -(\tau _{u} +\mu _{Au}) \bar{A}_u, \end{aligned}$$

12 $$\begin{aligned} \frac{d\bar{F}_u}{dt}= & {} \frac{\tau _{u}}{2}\bar{A}_u + \sigma \bar{F}_w -\mu _u \bar{F}_u, \end{aligned}$$

13 $$\begin{aligned} \frac{d\bar{A}_w}{dt}= & {} \left[ \frac{\rho _{ww} (\bar{F}_w^2 +\delta \bar{F}_w \bar{F}_u) }{\bar{F}}\right] \left( 1-\alpha \bar{A} \right) - (\tau _{w} +\mu _{Aw}) \bar{A}_w, \end{aligned}$$

14 $$\begin{aligned} \frac{d\bar{F}_w}{dt}= & {} \frac{\tau _{w}}{2}\bar{A}_w - \sigma \bar{F}_w - \mu _{w} \bar{F}_w. \end{aligned}$$

The modeling of *w*Au-*Wolbachia* transmission dynamics has not been done as this a distinction from other *Wolbachia* transmission models. Unlike the modeling work in Adekunle et al.^37^, apart from the non-induction of CI, we considered the loss of Wolbachia infections due to seasonal fluctuation in temperature, a key dynamics that is absent in *w*Au strain.

## Results

### Analysis of the model

The above general model (11)–(14) is parametrically adjusted to simultaneously accommodate *w*Au and *w*Mel *Wolbachia* strains. For the *w*Au-*Wolbachia* model, we set $\phi = \sigma = 0$ and for the *w*Mel-*Wolbachia* model, we set $\phi = 1$, $\sigma > 0$. The *w*Mel-*Wolbachia* model parameter adjustments correspond to the model studied in Adekunle et al.^37^.

Here, we want to analyse the general model(11)–(14) with arbitrary values of $\phi$ and $\sigma$ to enable comparison with *w*Au-*Wolbachia* and Adekunle et al. 2019^37^ models. Analysing the model for *w*Au, we have four steady states. The first steady state $e_1 = (0,0,0,0)$ indicates non-existence of mosquitoes. The second $e_2 = (A_u^*,F_u^*,0,0)$ signifies the steady state for the uninfected mosquito population only. The third $e_3 = (0,0,A_w^*,F_w^*)$ describes the equilibrium point for *w*Au-infected mosquitoes only. Lastly, the $e_4 = (A_u^*,F_u^*,A_w^*,F_w^*)$ is the equilibrium point for the co-existence of both uninfected and *w*Au-*Wolbachia*-infected mosquito populations.

#### Non-existence mosquito population, $e_1$

The equilibrium point $e_1$ is trivial and is not biologically realistic. However, we can gain some insights into the competitive model dynamics by examining the case where there is no interaction between the uninfected and *Wolbachia*-infected mosquitoes. In other words, we want to investigate how each population would behave in the absence of the other. In particular, we derive the reproduction number of the uninfected $R_{0u}$ and *Wolbachia*-infected $R_{0w}$ mosquito populations when they do not interact:

15 $$\begin{aligned} R_{0u}= & {} \frac{\rho _{uu}\tau _{u}}{2\mu _{u}(\mu _{Au}+\tau _{u})}, \end{aligned}$$

16 $$\begin{aligned} R_{0w}= & {} \frac{\rho _{ww}\tau _{w}}{2\mu _{w}(\mu _{Aw}+\tau _{w})}, \end{aligned}$$

where the factor of $\frac{1}{2}$ in $R_{0u}$ and $R_{0w}$ stems from the choice to set M = F^62^, i.e. $\psi = \frac{1}{2}$.

**Figure 2:**
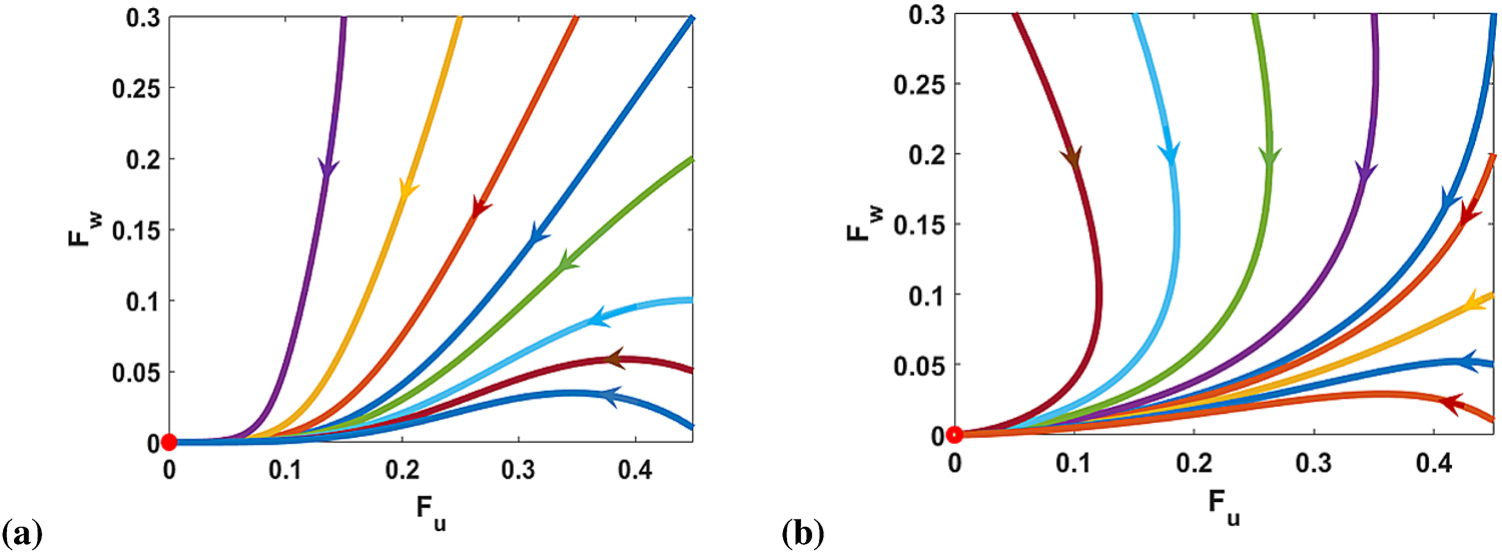
Graphs showing the system trajectories in the $(F_u, F_w)$ plane for (**a**) *w*Au ($\phi = \sigma = 0$) and (**b**) *w*Mel ($\phi = 1$, $\sigma =0.04$) *Wolbachia* models when $\max [R_{0u}, R_{0w}] < 1$. The red ball point indicates the point of stability, that is $(F_{u}, F_{w})=(0,0)$ representing mosquito extinction. We set $\rho _{uu}=0.01$ and $\rho _{ww}=0.1$. Other parameters used for these model simulations are provided in Table 2.

These reproductive numbers determine if the uninfected and *Wolbachia*-infected mosquito populations will die out or persist when there is no interaction. Specifically, if $R_{0u}<1$ and $R_{0w}<1$, then the two populations will die out (Fig. 2a). We observed in the decoupled case, the expressions for $R_{0u}$ and $R_{0w}$ are independent of the effects of CI $(\phi )$ and LWI $(\sigma )$ and are therefore equivalent for both the *w*Au and *w*Mel-*Wolbachia* strains (Fig. 2)^37^.

**Table 2:** Mosquito-Wolbachia model notations.

Parameters	Description	Values (*w*Mel)	Values (*w*Au)	Dimension	References
$\rho _{uu}$	Reproduction rate (egg laying rate) from mating between $F_{u}$ and $M_{u}/M_w$ mosquitoes	13	13	Eggs/day	^32,37,61^
$\rho _{ww}$	Reproduction rate (egg laying rate) from mating between $F_w$ and $M_{u}/M_w$ mosquitoes	10	10	Eggs/day	^30,37,61^
$\delta$	The proportion of *Wolbachia* infected eggs resulting from mating between $M_uF_w$ mosquitoes	0.95	0.95	Dimensionless	^30^
$\nu$	The proportion of *Wolbachia* infected eggs resulting from mating between $M_wF_w$ mosquitoes	1	1	Dimensionless	^37^
$\phi$	The CI induction	1	0	Dimensionless	^23^
$\psi$	Fraction of eggs that are male	0.5	0.5	Dimensionless	^37,62^
*K*	Carrying capacity of the aquatic stage *A*	$10^6$	$10^6$	aquatic mosquitoes	^37^
$\sigma$	Loss of *Wolbachia* infection (LWI)	0.04	0	day$^{-1}$	Assumed
$\tau _{u}$	Maturation rate of $A_{u}$ aquatic stage into adulthood (per capita)	0.11	0.11	day$^{-1}$	^30,61^
$\tau _{w}$	Maturation rate of $A_w$ aquatic stage into adulthood (per capita)	0.11	0.11	day$^{-1}$	^30,61^
$\mu _{Au}$	$A_{u}$ Aquatic stage mortality rate (per capita)	0.02	0.02	day$^{-1}$	^57^
$\mu _{Aw}$	$A_w$ aquatic stage mortality rate (per capita)	0.02	0.02	day$^{-1}$	^57^
$\mu _{u}$	$F_{u}$ adult mortality rate (per capita)	0.061	0.04316	day$^{-1}$	^23,37^
$\mu _{w}$	$F_w$ adult mortality rate (per capita)	0.068	0.08079	day$^{-1}$	^23,37^

#### Uninfected mosquito population, $e_2$

The uninfected-mosquito-only equilibrium point or *Wolbachia*-free equilibrium is

$$\begin{aligned} e_2 = \left( \frac{1}{\alpha }\left[ 1-\frac{1}{R_{0u}}\right] ,\frac{\tau _{u}}{2\mu _{u}\alpha }\left[ 1-\frac{1}{R_{0u}}\right] ,0,0\right) . \end{aligned}$$

For $e_2$ to exist, we require $R_{0u}>1$. In addition to the uncoupled reproduction numbers ($R_{0u}$ and $R_{0w}$) we also define the invasive reproduction number $R_{0w|u}$ which describes the average number of secondary offspring that will become *Wolbachia*-infected adults after introducing a single adult *Wolbachia*-infected mosquito into an established *Wolbachia* uninfected mosquito population.

To compute $R_{0w|u}$, we use the next generation matrix method^63^ to obtain

17 $$\begin{aligned} R_{0w|u}=\frac{\delta R_{0w}}{R_{0u}}, \end{aligned}$$

where we have substituted in the definition of $R_{0w}$ from Eq. (16). The invasive reproduction number $R_{0w|u}$ is the same for both *w*Au and *w*Mel-*Wolbachia* strains as that derived in Adekunle et al.^37^. This is because, the expression (17) clearly shows that the invasive reproductive number $R_{0w|u}$ is not dependent on the CI effect, $\phi$ or LWI, $\sigma$.

To check if the equilibrium point $e_2$ is stable, we compute the Jacobian of the system and evaluate it at $e_2$. In particular, letting $z_1 = (\mu _{Au}+\tau _{u})$ and $z_2 = (\mu _{Aw}+\tau _{w})$, yields

$$\begin{aligned} J_{e_2}=\begin{pmatrix} -z_1R_{0u}&{}\frac{\rho _{uu}}{R_{0u}}&{}z_1(1-R_{0u})&{}\frac{(1-\delta )\rho _{ww}}{R_{0u}}\\ \frac{\tau _{u}}{2}&{}-\mu _{u}&{}0&{}0\\ 0&{}0&{}-z_2&{}\frac{\delta \rho _{ww}}{R_{0u}}\\ 0&{}0&{}\frac{\tau _{w}}{2}&{}-\mu _{w} \end{pmatrix}. \end{aligned}$$

To obtain the characteristic equation of $J_{e_2}$, we have

$$\begin{aligned} |J_{e_2}-\lambda I|&=0, \end{aligned}$$

which becomes

$$\begin{aligned} (\lambda ^2 +k_1\lambda + k_2)(\lambda ^2 +l_1\lambda + l_2)&=0, \end{aligned}$$

where

$$\begin{aligned} k_{1}= & {} \mu _{u}+z_1 R_{0u},\\ k_{2}= & {} \mu _{u}z_1(R_{0u}-1),\\ l_{1}= & {} \mu _{w}+z_2,\\ l_{2}= & {} \mu _{w}z_2(1-R_{0w|u}). \end{aligned}$$

Therefore, $e_2$ is locally asymptotically stable if and only if $R_{0w|u}<1$ and $R_{0u}>1$ (Fig. 4). This is also consistent with the study in Adekunle et al.^37^ (See Table 3).

#### Wolbachia-infected mosquito population, $e_3$

The *w*Au-infected-only equilibrium point is $e_3 = \left( 0,0,\frac{1}{\alpha }\left[ 1-\frac{1}{R_{0w}}\right] ,\frac{\tau _{w}}{2\mu _{w}\alpha }\left[ 1-\frac{1}{R_{0w}}\right] \right)$. This again is consistent with Adekunle et al.^37^.

For $e_3$ to exist we require $R_{0w}>1$. By computation, the invasive reproductive number $R_{0u|w}$ with respect to uninfected mosquitoes is given as,

18 $$\begin{aligned} R_{0u|w}=\frac{R_{0u}}{R_{0w}}\left[ (1-\phi )+\frac{\rho _{ww}}{\rho _{uu}}(1-\delta ) \right] = \frac{c R_{0u}}{R_{0w}}, \end{aligned}$$

where $c = (1-\phi )+\frac{\rho _{ww}}{\rho _{uu}}(1-\delta )$. Clearly, $R_{0u|w}$ is dependent on $\phi$. For the *w*Mel-*Wolbachia* strain, i.e. $\phi =1$, $c = \frac{\rho _{ww}}{\rho _{uu}}(1-\delta )$ which is equivalent to that of Adekunle et al.^37^. However, for the *w*Au-*Wolbachia* strain, i.e. $\phi =0$, we have a modified expression of $c = 1+\frac{\rho _{ww}}{\rho _{uu}}(1-\delta )$ in Eq. (18) because we do not assume CI. Therefore, $c\ge 1$ for *w*Au-*Wolbachia* strain. Computing the Jacobian at $e_3$, we have:

$$\begin{aligned} J_{e_3}=\begin{pmatrix} -z_1&{}\frac{\rho _{uu}+(1-\delta )\rho _{ww}}{R_{0w}}&{}0&{}0\\ \frac{\tau _{u}}{2}&{}-\mu _{u}&{}0&{}0\\ z_2(1-R_{0w})&{}\frac{-(1-\delta )\rho _{ww}}{R_{0w}}&{}-z_2R_{0w}&{}\frac{\rho _{ww}}{R_{0w}}\\ 0&{}0&{}\frac{\tau _{w}}{2}&{}-\mu _{w} \end{pmatrix}. \end{aligned}$$

The characteristic equation of $J_{e_3}$ is then

$$\begin{aligned} |J_{e_3}-\lambda I|=(\lambda ^2 + m_1\lambda + m_2) (\lambda ^2 + n_1\lambda + n_2) = 0, \end{aligned}$$

where

$$\begin{aligned} m_{1}= & {} \mu _{u}+z_1,\\ m_{2}= & {} \mu _{u}z_1(1-R_{0u|w}),\\ n_{1}= & {} \mu _{w} + z_2 R_{0w},\\ n_{2}= & {} \mu _{w}z_2(R_{0w}-1). \end{aligned}$$

Therefore, $e_3$ is locally asymptotically stable if and only if $R_{0u|w}<1$ and $R_{0w}>1$ (see Fig. 4). The condition is equivalent to that found in^37^ with generalized expressions for $R_{0u|w}$ used in place of the reduced version presented there (see Table 3).

#### Coexistent mosquito populations, $e_4$

The equilibrium point for which both the uninfected and *Wolbachia*-infected populations coexist is

$$e_4 = (\frac{2\mu _u \beta F_w^*}{\tau _{u}},\beta F_w^*,\frac{2\mu _w F_w^*}{\tau _{w}},F_w^*)$$ where

$$\begin{aligned} F_w^*=\frac{1}{2\alpha }\left[ \dfrac{\left( 1-\frac{\xi }{R_{0w}}\right) \tau _u\tau _w}{(\mu _w \tau _{u}+\beta \mu _u \tau _{w})}\right] , \end{aligned}$$

$\beta = \dfrac{R_{0w}(R_{0u|w}-1)}{R_{0u}(R_{0w|u}-1)}$ and $\xi = \dfrac{(\beta +1)}{(\delta \beta + 1)}$. For $e_4$ to exist, we require $R_{0w}> \xi > 1$ and

(i) $$R_{0w|u},R_{0u|w}>1$$ or
(ii) $$R_{0w|u},R_{0u|w}<1$$.

The above conditions (i) and (ii) correspond to the cases for $\delta >\dfrac{1}{c}$ and $\delta < \dfrac{1}{c}$ respectively. Comparing these existence conditions with those found above for $e_2$ and $e_3$, we see that condition (ii) for the existence of $e_4$ matches the combined existence and local asymptotic stability condition for $e_2$ and $e_3$. In other words, $e_2$, $e_3$ and $e_4$ can coexist, while $e_1$ always exists (see Fig. 4).

To establish whether $e_4$ is stable or not, we compute the Jacobian $J_{e_4}$ evaluated at $e_4$ to obtain the following characteristic equation:

19 $$\begin{aligned} |J_{e_4}-\lambda I|:=\lambda ^4 +s_1\lambda ^3 + s_2\lambda ^2 +s_3\lambda + s_4 = 0. \end{aligned}$$

Let

$$z_3 = (\mu _u + \mu _w)$$, $$z_4 = (\beta \rho _{uu}+\rho _{ww})$$, $$z_5 = (\beta +1)\rho _{uu} +(1-\delta )\rho _{ww}$$, $$z_6 = 1+\beta (2+\beta \delta )$$,

$$z_7 = (\beta +1)^2\rho _{uu} +(1-\delta )\rho _{ww}$$, $$z_8 = (1+\beta (2+\beta \delta ))\rho _{uu} +(1-\delta )\rho _{ww}$$,

then we have:

$$\begin{aligned} s_{1}= & {} z_1+z_2+z_3+\alpha z_4F_w^*,\\ s_{2}= & {} \mu _u\mu _w + z_3(z_1+z_2 + \alpha z_4F_w^*) - \frac{\xi }{2 R_{0w}(\beta + 1)^2} (z_6\rho _{ww}\tau _w +z_7\tau _u),\\ s_{3}&=  {} \mu _u\mu _w (z_1+z_2+\alpha z_4F_w^*) +z_3 \left[ z_1 z_2 + \frac{\alpha }{\beta + 1}(z_1 (1+\beta \delta )\rho _{ww}+\beta z_2 z_5)F_w^* \right] \\&\quad -\frac{\xi }{2R_{0w}(\beta + 1)^3}\{\left[ (\mu _u +z_1)z_6+ z_8\alpha \beta F_w^*\right] (\beta + 1)\rho _{ww}\tau _w\\&\quad +\left[ \alpha \beta (1-\delta )z_5\rho _{ww}F_w^*+ z_7(\alpha (1+\beta \delta )\rho _{ww}F_w^*+(\mu _w +z_2)(\beta + 1))\right] \tau _u\},\\ s_{4}= & {} \mu _u\mu _w \left[ z_1 z_2 + \frac{\alpha }{\beta + 1}(z_1 (1+\beta \delta )\rho _{ww}+\beta z_2 z_5)F_w^* \right] - \frac{\xi }{2R_{0w} (\beta + 1)^2} \{[z_2z_6 \\&\quad +z_8\alpha \beta F_w^*]\mu _u\rho _{ww}\tau _w + [\alpha \beta (1-\delta )z_5\rho _{ww}F_w^*+ z_7(\alpha (1+\beta \delta )\rho _{ww}F_w^*+z_2(\beta + 1))]\mu _w\tau _u\\&\quad -\frac{\xi }{2R_{0w}} [z_8\rho _{ww}] \}. \end{aligned}$$

**Figure 3:**
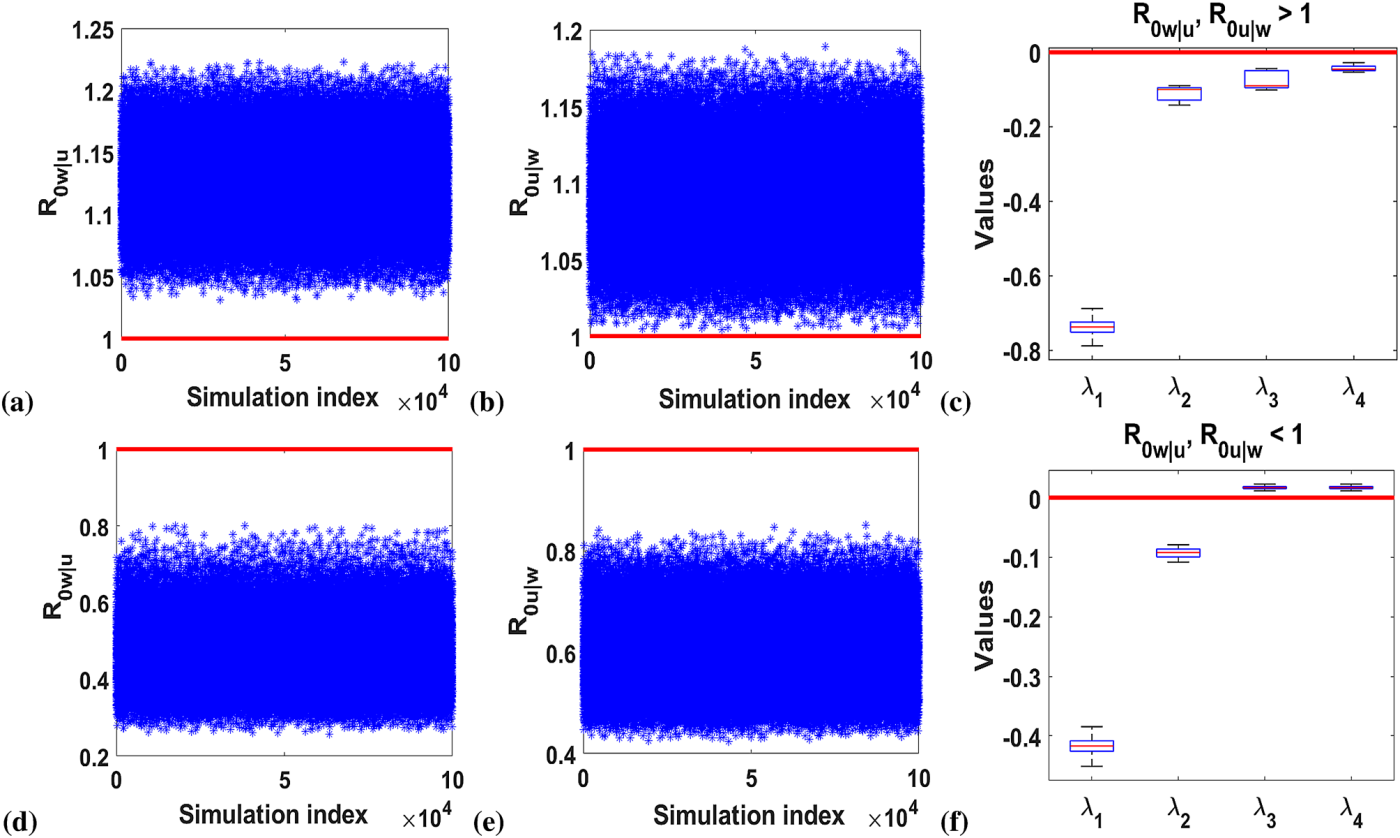
Graphs showing the numerical testing for the stability conditions (i) and (ii) and the real part of the eigenvalues’ distribution ($\lambda _1, \lambda _2, \lambda _3$ and $\lambda _4$) for $e_4$: (**a**,**b**) show that $R_{0w|u}, R_{0u|w} >1$ always hold. (**c**) shows the related distribution of the real part of the eigenvalues for condition (i). (**d**,**e**) show the condition $R_{0w|u}, R_{0u|w} <1$ always hold while (**f**) shows the corresponding distribution of the real part of the eigenvalues for condition (ii).

In order to establish the nature of the equilibrium point $e_4$, we performed numerical testing using the Monte Carlo method in^50^ to verify the conditions (i) and (ii) by computing the real part of the eigenvalues of the Jacobian matrix, evaluated at $e_4$. Simulation results are illustrated in Fig. 3.

Although the conditions (i) and (ii) indicated the existence of $e_4$, Fig. 3c showed that $e_4$ is locally stable for condition (i) as all the eigenvalues (real part) are negative ($\lambda _1, \lambda _2, \lambda _3,\lambda _4 <0$). Whilst Fig. 3f showed that $e_4$ is unstable for condition (ii) as two of the eigenvalues (real part) are positive i.e. $\lambda _3,\lambda _4 > 0$.

**Figure 4:**
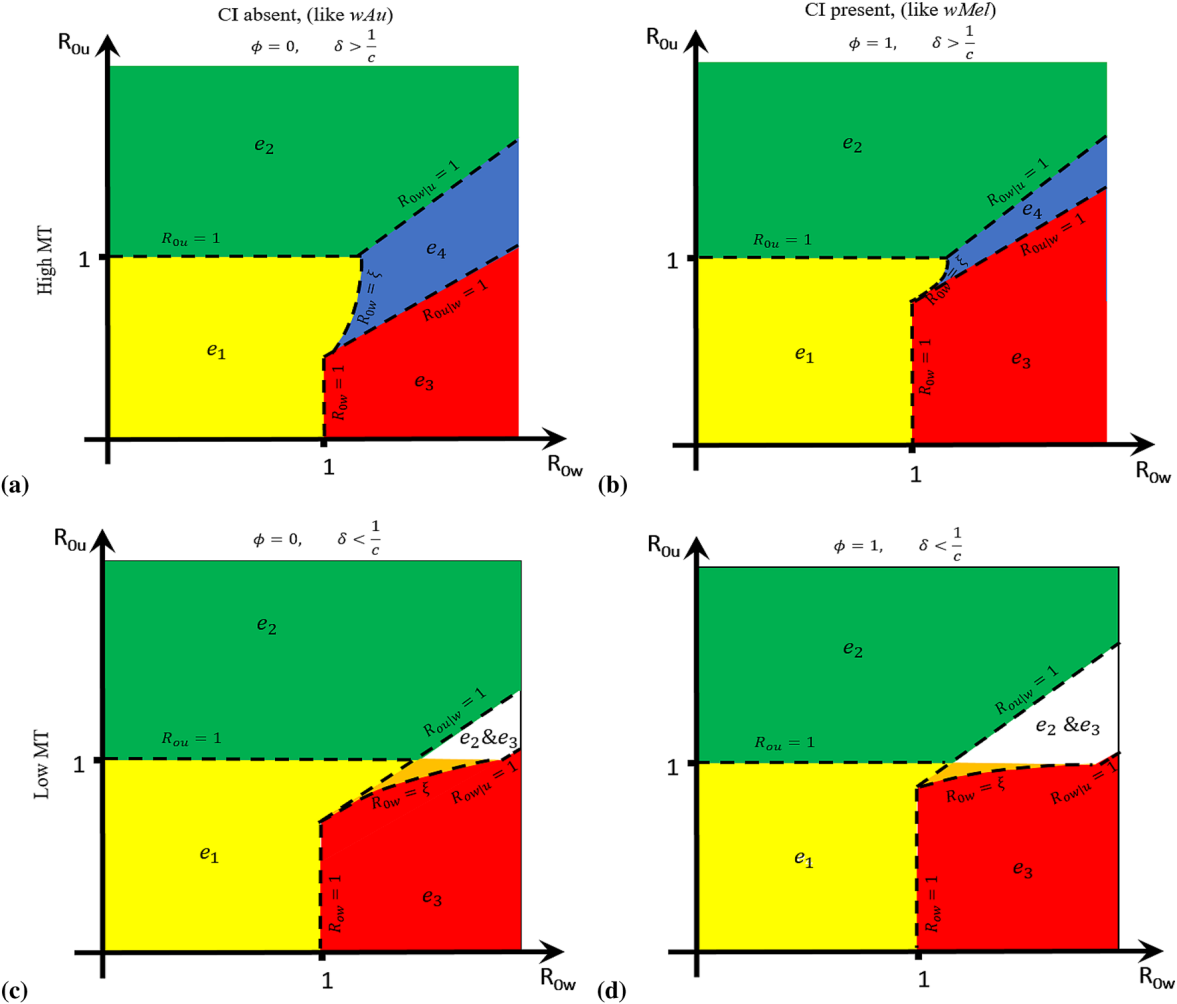
This graph shows the existence and local stability regions for the equilibrium points $e_1$–$e_4$ for the *Wolbachia* model (11)–(14) as a function of the $R_{0u}$ and $R_{0w}$ relating to the cytoplasmic incompatibility (CI), $\phi$ and maternal transmission (MT), i.e. magnitude of $\delta$ and $\frac{1}{c}$. The yellow shaded region indicates the local stability of $e_1$ equilibrium. The green shaded area illustrates the local stability for the *Wolbachia*-free equilibrium point ($e_2$). $e_3$ is locally stable at the red shaded part. The blue region indicates the coexistence local stability $e_4$. The white region shows the existence of $e_2, e_3$ and $e_4$ and local stability of $e_2$ and $e_3$ equilibrium points. And the orange region describes the existence and local stability of $e_1$ and $e_3$. For $\delta >\frac{1}{c}$; (**a**) describes $\phi =0$ as the boundary $R_{0w|u}=1$ sits above the boundary $R_{0u|w}=1$ and the arc $R_{0w}=\xi$ . The co-existent equilibrium $e_4$ (blue), always sits in the region between these three boundaries because $R_{0w|u}>1$, $R_{0u|w}>1$ and $R_{0w}>\xi$. If $R_{0w}<\xi$, then $e_1$ becomes stable (yellow). (**b**) describes similar conditions as in (**a**) but for $\phi =1$. We observed that the boundary $R_{0u|w}=1$ shifts up while $R_{0w|u}=1$ remained stationary to accommodate more $e_3$. For $\delta <\frac{1}{c}$; (**c**) describes $\phi =0$ as the relative position of $e_4$ boundaries in (**a**) flips so that boundary $R_{0u|w}=1$ sits above boundary $R_{0w|u}=1$ and the arc $R_{0w}=\xi$. Then, $R_{0w|u}<1$ and $R_{0u|w}<1$ and $R_{0w}>\xi$ shows the co-existence of $e_2$ and $e_3$ (white). However, $e_2$ and $e_3$ are locally stable in the white region as $R_{0w}>1$ and $R_{01}>1$. For $R_{0u}<1$, $e_2$ and $e_4$ do not exist, only $e_1$ and $e_3$ do and if $R_{0w}>\xi$, $e_1$ and $e_3$ are locally stable (orange) and if $R_{0w}<\xi$, only $e_3$ becomes stable (red). (**d**) describes similar conditions as in (**c**) but for $\phi =1$. It was observed that the boundary $R_{0u|w}=1$ shifts up reducing the region of stability for $e_2$.

Numerically, we illustrated the existence and stability regions for $e_4$ in Fig. 4 for the two conditions (i) and (ii) relating to CI and maternal transmission (MT).

Following a modeling study of *Aedes aegypti* mosquitoes and normal *Wolbachia* (in the presence of CI only) interaction analyzed by Ferreira et al.^64^, three equilibrium points: trivial ($q_1$); uninfected only ($q_2$); and coexistence ($q_3$), were obtained. However, the *Wolbachia*-only equilibrium point was not computed. The established local stability conditions for $q_1$ and $q_2$ correspond to that of the *w*Mel-like *Wolbachia* conditions for $e_1$ and $e_2$ respectively. For coexistent populations to persist, the reproductive number for infected mosquitoes only, $R_i$ must be greater than 1 and $R_i > R_u$, where $R_u$ is the reproductive number for wild-type mosquitoes only. The model^64^ also described the fitness parameter space between $R_u$ and $R_i$, showing the change in extinction and persistence of the three equilibria when there is an increase in the initial population proportion of the *Wolbachia*-infected mosquitoes. Our model showed the changes in the no-mosquito, wild-type only, *Wolbachia*-only and coexistence population persistence and extinction in the presence and absence of CI with high and low maternal transmission (MT).

**Figure 5:**
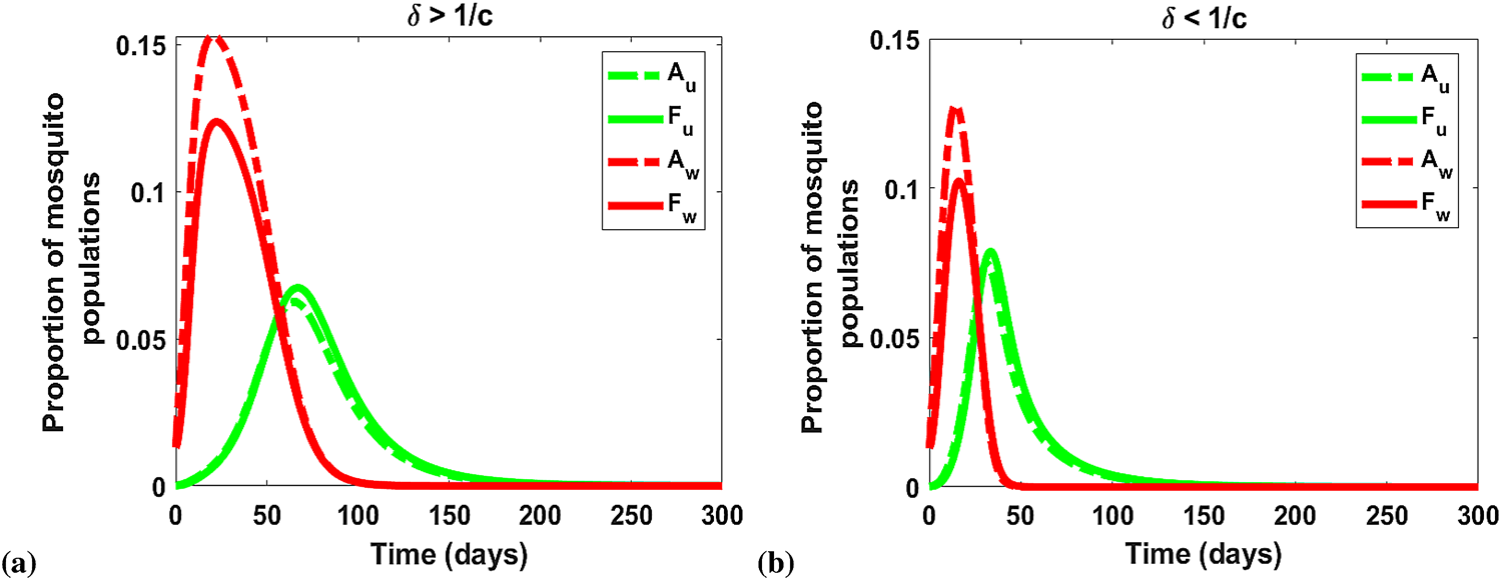
Graphs showing the local stability for $e_1$ relating to the magnitude of $\delta$ and $\frac{1}{c}$. The initial conditions for the state variables are $A_u(0)=0.00015$, $F_u(0)=0.00013$, $A_w(0)=0.013$, $F_w(0)=0.013$. We set $\rho _{uu}=1, \rho _{ww}=2.8571, \tau _u =\tau _w =1, \mu _{Au}=\mu _{Aw}=0.2, \mu _u=0.4630, \mu _w=0.6161$. (**a**) For $\delta >\frac{1}{c}$, where $\delta =0.4, c=2.7143, R_{0u}=0.8999, R_{0w}=1.9322, R_{0u|w}=1.2641, R_{0w|u}=0.8588$. (**b**) For $\delta <\frac{1}{c}$, where $\delta =0.2, c=3.2857, R_{0u}=0.8999, R_{0w}=1.9322, R_{0u|w}=1.5303, R_{0w|u}=0.4294$. The equilibrium point $e_1$ is locally stable if $R_{0u}<1, R_{0w}>1$, $R_{0w|u}<1$ and $R_{0u|w}>1$.

Figure 4 illustrates the existence and local stability regions for the equilibrium points $e_1$, $e_2$, $e_3$ and $e_4$ with respect to the reproduction numbers $R_{0u}$ and $R_{0w}$ as well as the relative magnitude of $\delta$ and $\frac{1}{c}$. For $\delta > \frac{1}{c}$ (high MT), Fig. 4a,b describe the dynamics for $\phi =0$ (CI absent) and $\phi =1$ (CI present) respectively. Within the subset of the yellow region of these figures bounded by $R_{0u}=1, R_{0w}=1$, and $R_{0w}=\xi$ we find that only $e_1$ and $e_3$ exist. Since $e_3$ is unstable in this region, we expect the system trajectories to tend to the no-mosquito equilibrium $e_1$. This was confirmed through numerical simulations shown in Fig. 5a. For the existence of $e_4$ we require $R_{0u|w}>1$, $R_{0w|u}>1$ and $R_{0w}>\xi$ for stability (within the blue region). But if $R_{0w}<\xi$, $e_1$ is stable (yellow).

For $\delta < \frac{1}{c}$ (low MT), Fig. 4c,d portrayed the regions of stability for $\phi =0$ and $\phi =1$ respectively. The conditions $R_{0u}<1, R_{0w}>1$, and $R_{0u|w}>1$ project the trajectiory to tend to $e_1$ (see Fig. 5b). In the orange region, $e_1$ and $e_3$ exist and are simultaneously locally stable as $R_{0u|w}>1$ and $R_{0w}>\xi$. In addition, we have that $e_4$ exists where $R_{0u|w}<1$ and $R_{0w|u}<1$ (condition (ii)). With these conditions, $e_4$ exists together with $e_2$ and $e_3$ (white region). In this white region, $e_2$ and $e_3$ are locally stable even as $R_{0u}>1, R_{0w}>1$ but $e_4$ is unstable. Also, $e_1$ exists when $R_{0w}>1$ and $R_{0u}<1$ because the local stability of other equilibrium points is violated with these conditions. When $R_{0u|w} > 1$ but $R_{0u} < 1$ and $R_{0w} > 1$, the only stable outcome is the mosquito-free (no-mosquito) equilibrium $e_1$. This occurs when $R_{0u}$ is less than but still close to one. In this region, uninfected mosquitoes are capable of dominating initially when introduced into a *Wolbachia* saturated equilibrium because imperfect maternal transmission achieves $R_{0u|w} > 1$. This competitive advantage drives out the *Wolbachia* infected mosquitoes leaving uninfected mosquitoes only, which then are unable to sustain their population because $R_{0u} < 1$ (Fig. 5).

With the rate of high maternal transmission (MT) in the absence of CI (like-*w*Au), the reproductive advantage favours the production of uninfected mosquito offspring as it tends to accommodate more coexistent mosquito populations with wild-type than *w*Mel-like strain (presence of CI) due to the presence of CI (Fig. 4a,b). Whilst, with a low MT rate, the CI presence or absence would favour *Wolbachia*-infected mosquitoes or uninfected mosquitoes respectively. In other words, the coexistent equilibrium point is unstable for the two mosquito populations as these conditions are equivalent to the local stabilities of both *Wolbachia*-free and *Wolbachia*-only equilibrium points (Fig. 4c,d). If $R_{0w} < \xi$, the system trajectories tend to the no mosquito equilibrium $e_1$.

The conditions for the local stability of all equilibrium points are shown in Table 3 below.

**Table 3:** Expressions for the condition for stability associated with the equilibrium points.

Equilibrium points	Conditions for stability	
	wMel^37^	wAu
(i) No mosquitoes $(e_1)$	$R_{0u}<1$ and $R_{0w}<1$	$R_{0u}<1$ and $R_{0w}<1$
(ii) Uninfected mosquitoes only $(e_2)$	$R_{0w|u}<1$ and $R_{0u}>1$	$R_{0w|u}<1$ and $R_{0u}>1$
(iii) *Wolbachia*-infected mosquitoes only $(e_3)$	$R_{0u|w}<1$ and $R_{0w}>1$	$R_{0u|w}<1$ and $R_{0w}>1$
(iv) Both mosquitoes $(e_4)$	$\delta <1$, $\mu _u<\delta \mu _w$, $R_{0w}>1$ and $R_{0u}>1$	$R_{0w|u}>1$, $R_{0u|w}>1$, $R_{0w}>1$ and $R_{0u}>1$

### Sensitivity analysis of *Wolbachia* model

To carry out the sensitivity analysis we investigate the model robustness due to uncertainties associated with parameter value estimations. In other words, we examine how senitive the invasive reproductive numbers are with respect to these parameters. This in turn, gives insight on influential parameters and their impact in reducing (or increasing) mosquito-type populations. To carry out this, we compute the normalized sensitivity indices of the invasive reproduction numbers with respect to the parameters used in the model.

#### Definition

The normalized forward sensitivity index of a variable *v* with respect to parameter *w* is defined as:

20 $$\begin{aligned} \Lambda _w = \dfrac{\partial v}{\partial w} \times \dfrac{w}{v}. \end{aligned}$$

**Figure 6:**
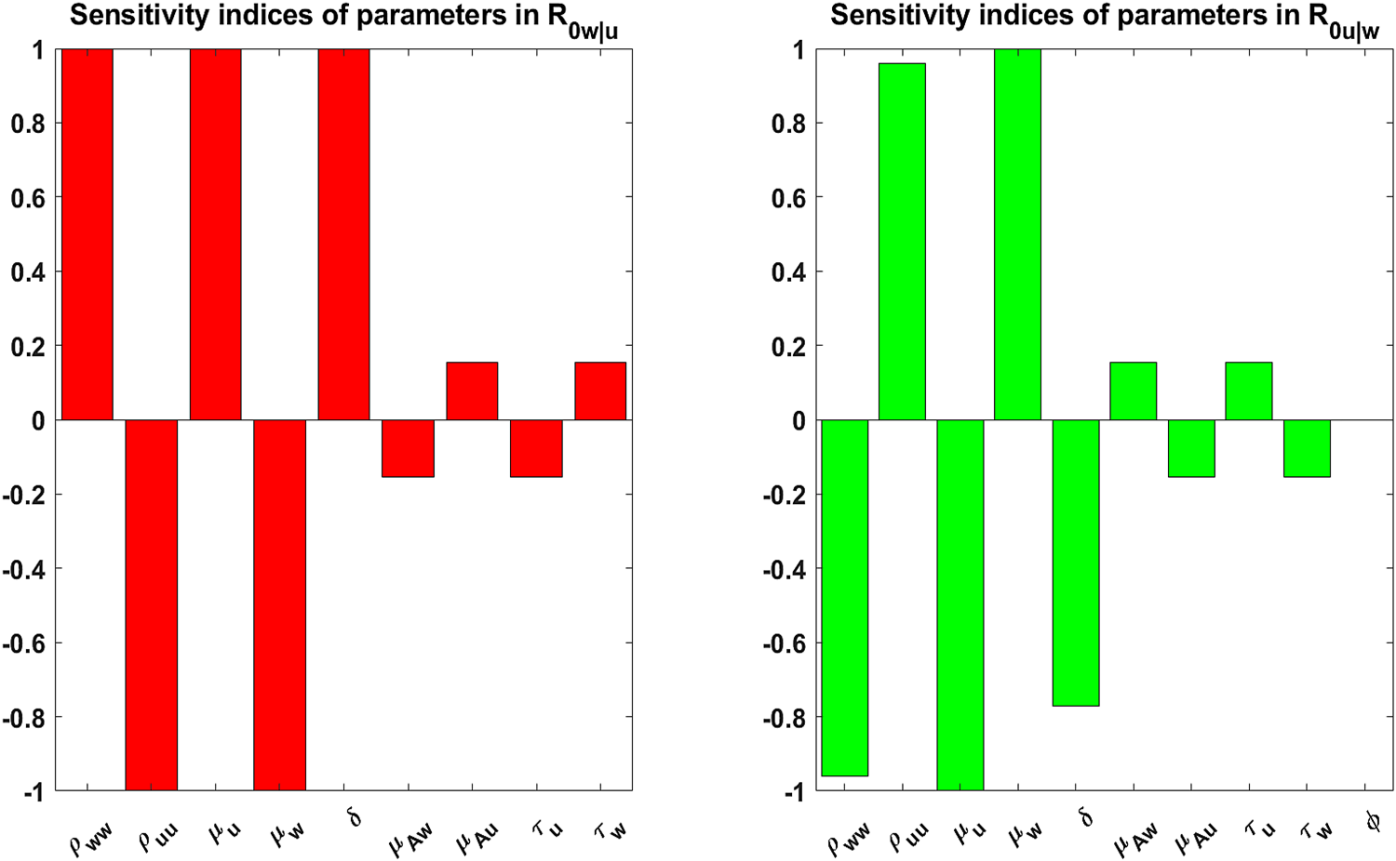
Plots showing the sensitivity indices of $R_{0w|u}$ and $R_{0u|w}$ the model parameters.

Using the above formular (20), we contruct the following plots in Fig. 6.

From Fig. 6 and using the baseline parameter values for the *w*Au-*Wolbachia* strain in Table 2, it is clear that the reproductive and mortality rates for both wild-type ($\rho _{uu}, \mu _u$) and *w*Au-*Wolbachia*-infected ($\rho _{ww}, \mu _w$) mosquitoes and the proportion of *w*Au-*Wolbachia*-infected offspring ($\delta$) have the most sensitivity in the invasive reproductive numbers $R_{0w|u}$. Whilst for $R_{0u|w}$, $\mu _u$ and $\mu _w$ are the most sensitive parameters. Hence for both invasive reproductive numbers, the most sensitive parameters are $\mu _u$ and $\mu _w$. This demonstrates that an increase (or decrease) in the mortality rate of *w*Au-*Wolbachia*-infected mosquitoes by 10% will decrease (or increase) $R_{0w|u}$ by 10%.

### Does CI $(\phi )$ outweigh the LWI $(\sigma )$?

For most *Wolbachia* strains except *w*Au, the mating between uninfected female and *Wolbachia*-infected male mosquito crosses generates no viable offspring. However, *Wolbachia*-infected mosquitoes tend to lose their *Wolbachia* infection and lower their maternal transmission rate at high temperature ($27{-}37^\circ {\text{C}}$)^23^. With the effect of climate change gradually increasing the temperature by the day, *Wolbachia* strains with moderate or high temperature sensitivity such as *w*Mel may not be able to fully maintain a sufficient frequency level to invade the mosquito population.

**Figure 7:**
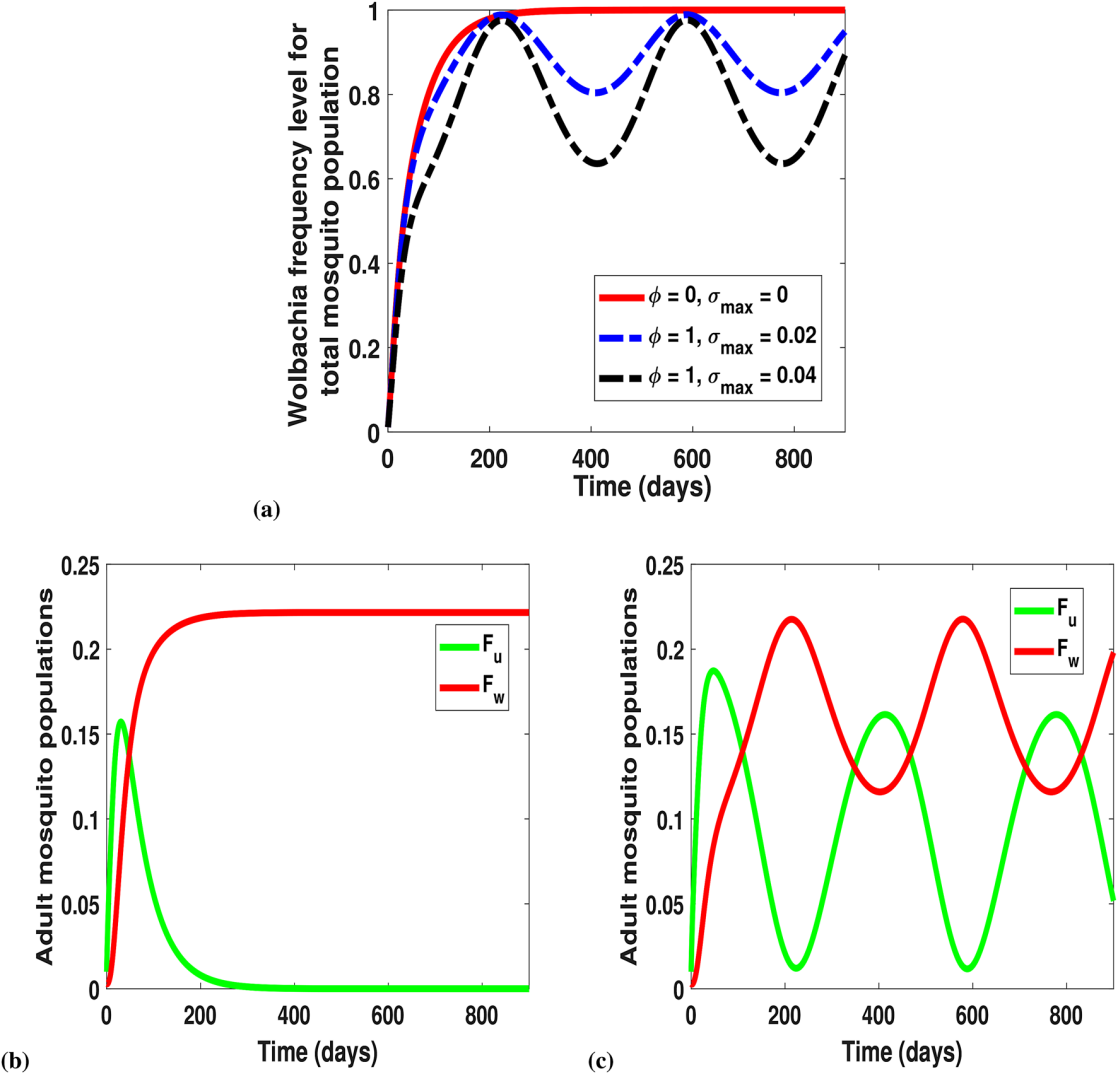
(**a**) Effect of CI induction $\phi$ and LWI $\sigma (t)$ on the *Wolbachia* frequency level. The initial conditions for the state variables are $A_u(0)=0.25$, $F_u(0)=0.01$, $A_w(0)=0$, $F_w(0)=0.003$. The red line indicates *Wolbachia* retainment as $\phi =0$ (no CI induction) and $\sigma _{max}=0$ (no LWI) which are features of *w*Au-*Wolbachia* strain. The blue and black dashed lines (for *w*Mel-*Wolbachia* strain) illustrate CI induction and LWI i.e $\phi =1$ for $\sigma _{max}=0.02$ and $\sigma _{max}=0.04$ respectively. Parameters for $e_3$ were used in these simulations. (**b**) Shows the dominance of *w*Au-*Wolbachia* infected $F_w$ to uninfected $F_u$ adult mosquitoes due to the retainment of *Wolbachia* infections (not affected by seasonal varying LWI). The *w*Au-*Wolbachia*-infected mosquitoes dominates when there is no CI $\phi =0$ and LWI $\sigma _{max}=0$ (red line). **(c)** For *w*Mel-*Wolbachia*-infected mosquitoes, the effect of seasonal varying loss of *Wolbachia* infection is shown as infections rise and drop continuously due to LWI $\sigma _{max}=0.04$ and CI induction $\phi =1$.

In our general *Wolbachia* mathematical model, we describe a modified version of Adekunle et al.^37^. This modification accommodates parameter adjustments for novel *w*Au and *w*Mel-*Wolbachia* strains. For *w*Au, our mathematical model showed that despite the production of mosquito offspring due to CI absence, the invasive reproduction number due to infected mosquitoes $R_{0w|u}$ remains unchanged compared to the case where CI is present, as with the *w*Mel-like strain^37^. This further strengthened the fact that CI (inclusion or exclusion) does not guarantee *Wolbachia* mosquitoes’ persistence. Also, the invasive reproduction number due to uninfected mosquitoes expression $R_{0u|w}$ for *w*Au is similar to *w*Mel, except that the expression depends on CI the effect. This is because, the mosquito gender crosses due to non-induction of CI for *w*Au, i.e. $F_uM_w$, generates uninfected offspring with perfect maternal transmission while *w*Mel does not. The chances of establishing *Wolbachia* infected mosquitoes are lower when CI is ineffective compared to when it is induced. That is, for cytoplasmic inducing *w*Mel-*Wolbachia* mosquitoes, the effect of LWI outweighs CI effect as mosquitoes still lose their infections (Fig. 7). However, *w*Au-*Wolbachia* infection retainment (no LWI) in mosquitoes has shown high level of maintaining the *Wolbachia* frequency in the absence of CI in mosquitoes (Fig. 7). This suggests that the LWI effect outweighs CI.

The LWI rate $\sigma (t)$ which is dependent on the seasons of the year can be modeled by a sinusoidal equation:

21 $$\begin{aligned} \sigma (t) = \frac{\sigma _{max}}{2}\left[ 1+\cos \left( \dfrac{2\pi t}{365}-\mathscr {C}\right) \right] \end{aligned}$$

where $\sigma _{max}$ is the maximum value of the seasonal variation in LWI, and $\mathscr {C}$ is the phase shift which aligns the model with the seasonal change.

The effects of CI ($\phi$) and LWI ($\sigma (t)$) as features of *w*Au and *w*Mel *Wolbachia* strains are shown in Fig. 7. For the total mosquito population, *w*Au-infected mosquitoes ($\phi =0, \sigma _{max}=0$) reach the maximum frequency after approximately 250 days. To see the effect of CI induction and slight LWI i.e. $\phi =1$, $\mathscr {C}=0.25$, for $\sigma _{max}=0.02$ and $\sigma _{max}=0.04$, the *Wolbachia* frequency level oscillates between (0.8 and 1) and (0.6 and 1) respectively. That is, there is a 20% and 40% drop in the frequency level of *Wolbachia* when $\sigma (t)$ is at $\sigma _{max}=0.02$ and $\sigma _{max}=0.04$ respectively. This showed that, despite CI induction, LWI reduced the contribution of CI to the *Wolbachia* invasion (Fig. 7a). Therefore, the LWI gains highly outweigh the CI effect. By this, our analysis suggests that an increase in LWI in the presence of CI results in a drastic decrease in the *Wolbachia* frequency level (Fig. 7a). On the other hand, Fig. 7b showed the effect of LWI $\sigma (t)$ and CI $\phi$ with respect to the competitiveness between $F_u$ and $F_w$. We observed that the $F_w$ population dominates the $F_u$ when there was no CI induction and *Wolbachia* infection is retained, that is, $\phi =0, \sigma _{max}=0$ (Fig. 7b). However, if CI induction occurs with loss of *Wolbachia* infections, then the seasonal varying effect occurs as seen in Fig. 7c.

## Discussion

In this work, we modelled and investigated a general *Wolbachia* model that contained the transmission dynamics of *w*Au and *w*Mel *Wolbachia* strains in *Aedes* mosquitoes as special cases. These transmission dynamics described the competition between the novel *w*Au-*Wolbachia* infected *Aedes* mosquitoes and wild-type mosquitoes and compared the dynamics with the invasive properties of the popular *w*Mel-*Wolbachia* infected mosquitoes. We first derived the *Wolbachia* infection-status reproduction numbers for our *w*Au-*Wolbachia* model and used them to establish the conditions for the local stability of the equilibrium points for the *w*Au-*Wolbachia* invasive model. The reproduction number associated with the uninfected mosquitoes shows the reproductive advantage that the wild type has over the *w*Au strain. The comparison of the *w*Au-*Wolbachia* model (CI and LWI absent) and *w*Mel-*Wolbachia* model (CI and LWI present) showed that the *w*Au strain has the potential of compensating for the undesirable features of the *w*Mel strain.

Additionally, this study has reviewed the main features of different *Wolbachia* strains (Table 1) and shown that the *w*Au *Wolbachia* strain is a promising candidate for efficient *Aedes*-borne arboviral transmission control. Moreover, we analyzed the system dynamics of a general *Wolbachia* invasion model and determined the regions of local stability for each of the identified equilibrium points, highlighting the regions in parameter for which *Wolbachia*-infected mosquito populations persist or go extinct. This work modelled the general *Wolbachia* dynamics which can accommodate various *Wolbachia* characteristics regarding the presence or absence of CI and seasonal changes, unlike Adekunle et al.^37^, which considers only the presence of CI. We also investigated the advantages gained from CI and LWI. This study has demonstrated that despite the absence of CI, the *Wolbachia* frequency level will drop as much as tenfold of the percentage of *Wolbachia* infection lost. We showed that the advantage of *Wolbachia* retainment in mosquitoes strongly outweighed the negative impact of CI indicating *w*Au *Wolbachia* strains may be suitable for arboviral control. Therefore, this modeling work contributes to the previous studies^37,54,57,64,65^ and helps close the gap between ways of maintaining the *Wolbachia* frequency levels in the absence of LWI and CI.

One implementation question for using the *w*Au strain as a replacement of the *w*Mel strain is whether the *w*Au strain is self-sustaining, given that it does not induce CI. In this work, the equilibrium points for the *w*Au-*Wolbachia* model are the same as that for the *w*Mel-*Wolbachia* model except that stricter conditions are required to satisfy the *w*Au-*Wolbachia* model equilibrium points. These more stringent conditions translate to additional resources such as the continuous introduction of a larger scale of *w*Au-infected mosquitoes to ensure replacement^66^. Thus, the *w*Au strain is a promising alternative strain as it does not suffer from LWI due to high weather temperature and is highly effective in preventing the transmission of the arbovirus^23,39,67^. Otherwise, combining the two strains may also be a good strategy.

There are limitations associated with any mathematical modeling work, and this study is not exempted. We first assumed the same mosquito gender ratio and expected this proportion to be constant over time. This assumption may be true in a laboratory setting^62^, but not necessarily true in a natural mosquito habitat. However, similar conclusions are expected to be reached as the *Wolbachia* model reduction accurately reproduces the dynamics of the full system^68^. Secondly, we assumed that the absence of CI implies that cross mating resulted in offspring that are uninfected. This may not be true as a small proportion of the offspring may be *Wolbachia* infected^23^. If that is the case, then it means that lesser resources will be required to use the *w*Au strain as a *Wolbachia*-based control strategy. Lastly, we assumed the seasonality affects the associated parameters for the *w*Mel dynamics. However, for the *w*Au strain, it is not affected by seasonality as *w*Au-*Wolbachia* infections are retained at high temperature.

Although several studies^22,38,42,57^ have demonstrated that CI drives the persistence of *Wolbachia*-infected *Aedes* mosquitoes, these studies neglected the impact of *Wolbachia* loss in mosquitoes. The CI drive has been shown in four mating lines (see Fig. 1) involving a *Wolbachia*-transinfected *Aedes* mosqiutoes mating with wild-type mosquitoes. One of the mating lines for which *Wolbachia*-infected male and uninfected female mosquitoes produced no viable offspring (via CI) truncates the uninfected offspring from being produced as infection is maternally transmitted. With the exception of the mating between the uninfected male and female mosquito line, all other mating lines produce *Wolbachia*-infected offspring leading to persistence. In addition, high temperature affects these *Wolbachia*-infected mosquitoes as they lose their infection due to the unfavourable weather conditions. However, mosquitoes infected with the *w*Au-*Wolbachia* strain have been shown to not only block arboviral transmission efficiently, but also retain the *Wolbachia* infection at typically unfavourable high temperatures. This retainment of infection in mosquitoes strongly outweighed the absence of CI for the *w*Au strain in the establishment and dominance of *w*Au-*Wolbachia* infected mosquitoes.

While vaccine implementation may have been highly effective on dengue seropositive persons in high transmission areas^11,12^, the introduction of *Wolbachia*-infected mosquitoes in low and moderate arboviral endemic areas has also effectively shown successful reduction in dengue burden^43,51,59,69^. Given that these two strategies could reduce the transmission of *Aedes*-borne diseases, in particular, dengue depending on the transmission level, a modeling study by Ndii^70^ proposed the use of these combined strategies and compared their effectiveness. The author showed that, *Wolbachia* performs better in the presence of low vaccine efficacy, but is outperformed otherwise^70^. Therefore combining the two strategies may be useful, however understanding both the temperature and seasonality effects on *Wolbachia* intervention programs, and serotypic differences relating to cross-protective immunity to investigate vaccine efficacy is necessary for the reduction and control of *Aedes*-borne arboviral disease transmission.

In conclusion, we have shown that the *w*Au-*Wolbachia* strain could be effective in controlling arbovirus transmission, as its advantages in terms of *Wolbachia* infection retention in mosquitoes may outweigh the absence of CI. This could prove even more promising, especially as the temperature increases due to climate change. Although *w*Mel and *w*AlbB-*Wolbachia* strains only have been rolled out in natural mosquito habitats in replacement programs, combining these strains with *w*Au is worth exploring.

## Data Availability

No datasets were generated or analysed during the current study.
